# Some Observations upon Hereditary Tendencies and Transmissions

**Published:** 1881-09

**Authors:** Roger Marvin Griswold

**Affiliations:** Member and Fellow of the Connecticut State Medical Society, and of the American Medical Association, North Manchester, Ct.


### Independent Practitioner.

Vol. II.——*September*, 1881.——No. 9.

ARTICLE I.

### SOME OBSERVATIONS UPON HEREDITARY TENDENCIES AND TRANSMISSIONS.

By ROGER MARVIN GRISWOLD, M. D.,
Member and Fellow of the Connecticut State Medical Society, and of the American Medical Association, North Manchester, Ct.

The subject of hereditary tendencies and transmissions in the human species presents a field so vast, and so open to inexhaustible research, that the limited space allowed will permit of but a few general thoughts upon the subject, and, in doing this, I cannot be expected to present to the readers of the PRACTITIONER anything either very new or wonderfully original, but simply to call their attention a little more closely to this subject, that in their daily search for the *causes* which underlie diseases they shall not overlook this often most potent of all causes, which is so frequently a predominating influence in a very large number of the troubles we are called upon to treat.

The observation of the transmission of personal peculiarities and conditions antedates history, and is probably coeval with the existence of humanity itself ; for so obvious a phenomenon could not long escape the observation of ordinary intelligence.

" Like father, like son," is so often true that we may be certain it was easy observed, and when we come out of the mists of pre-historic ages into the dim light of the first records of humanity, we begin to find notice taken of it. Hippocrates is the first writer who has left behind him any records of his observations upon this subject, and he gives it extended notice, and places much emphasis upon its certain action, especially in morbid tendencies.

Tacitus, about A. D. 80, and Lucretius, at a somewhat later period, also refer to it, and more modern writers have contributed largely to the literature of the subject. Mercatus, a little more than three centuries ago, published some very valuable observations relative to the matter. His investigations led him to believe that "the quality, character, essential structure, proportion or disproportion, whether of one or several members, as it appeared in the offspring, was engendered in the parent, and are similar affections or defects to those existing in the parents or grandparents ; that nature employs the same instrumentality in transmitting them, whatever may have been their origin, and that children are born similar to their parents, and with like blemishes."

At a much later date Portal writes as follows: "Not only are marks of the body transmitted from father to son, but also a resemblance of temper, complexion and imitations of the mind."

It is within the knowledge of all that children often possess the form and features of one or the other of their parents, or a combination of both. There are family likenesses sometimes so strongly marked by peculiar confirmation of form and feature, that we easily distinguish a son by his likeness of his father or mother, or by his resemblance to a brother, and even recognize the peculiar features in persons of the same blood, though more distantly removed.

We observe also that in isolated districts where custom, prejudice, or other favoring circumstances have tended for a long time to restrict marriage with people of other countries, a striking peculiarity of figure, or face, or both, becomes developed, as also certain mental and moral characteristics. Proceeding from communities and clans to nations we observe the same results. The physiogomy of the Jews, who, more than any other of the Semitic race, have preserved themselves unadulterated from the earliest periods of recorded history, is the same to-day, as when during the captivity, thousands of years ago, it was recorded in stone in the land of the Pharoahs.

We observe not only how the peculiar physical conformation and color of a race descend from generation to generation, but we also find special congenital *peculiarities* occasionally transmitted.

A German author mentions two families who for several generations were distinguished by six fingers on each hand, six toes on each foot.

Hallen records a family where the father, grandfather and great-grandfather were all web-footed. There lives at the present time in one of the inland cities of New York a man, whose father and grandfather, like himself, were born and continued without finger or toe nails. An Italian author mentions the case of a Maltese man and wife who were both web-footed, and who transmitted the peculiarity to each one of their sixteen children. Zera Colburn, the distinguished mathematician, had six fingers on each hand, which peculiarity he inherited from his father. The thick lips of the imperial house of Austria, noticeable at the present day, are descended for many generations from the marriage of the first Maximilian with Mary of Burgunda.

If in addition to facts presented to us by writers upon the subject, and the certain deductions from our own observations in daily life, we turn to the brute creation for an analogy to supplement our views, we find it in many domesticated animals. Every farmer knows that certain breeds of stock inherit certain qualities of physical conformation, and of adaptation to certain uses. Every horseman knows that one breed of horses develops especial fitness for the fields, another for the turf, and he is ignorant indeed who expects to develop 2.20 out of ever so many generations of English dray horses, or anticipates the production of a heavy draft horse from a breed of pure Morgan, or a Kentucky runner.

If particular points are desired, those points must be especially cultivated and bred to,

And here allow me to say, that however mortifying it may be to the boasted superiority of the human race over all others of the animal creation, the *physical* man is an *animal*, and nothing more, and subject to the same immutable laws that govern the inferior species about him. He is built up of the same materials, under the operation of the same general laws, and springs from an original cell that has no *recognizable* difference from that from which springs the meanest of God's creatures. I say "no recognizable" difference, for, although beyond human ken, there must be something, an imponderable force, that *does* determine the one cell into a worm, and the other into that animate existence "a little lower than the angels," yet human science has as yet failed to detect the difference.

In the laws of man's propagation, his continuation and decay, the fundamental principles are the same, immutable and unchangeable, and however it may be in the moral world, in the physical, these laws if broken, are certain to verify the truth of the declaration that "the sins of the fathers shall be visited upon the children, even unto the third and fourth generation." Yes, and so much further that it is impossible for us to determine. Each generation has great power over the natural gifts, and physical infirmities of those generations which follow, and it is a duty we owe to humanity to investigate the range of that power; and to exercise it in a way that shall be most advantageous to those that follow after us. Galton, in his admirable work on "Hereditary Genius," advances the thought that the often expressed idea that babies are born pretty much alike, and that the sole agencies in creating differences between boy and boy and man and man are steady application and moral effort, is great nonsense. I acknowledge the use of education and social influences in developing the powers of the mind, just as I acknowledge the influence of labor in developing the muscles of the blacksmith's arm, and no further. The blacksmith or the gymnast may labor or train until they have developed their muscles to the greatest extent, but they reach a limit, and some day, a man who in all his life has never done so much work as have they in one week, by the exertion of his mere *natural strength*, performs feats which they never dreamed of doing, and this is similar to the experience of everyone in the working of his mental powers. The eager boy, when he first goes to school and confronts intellectual difficulties, is astonished at the progress he makes. He wonders at his increasing capacity for application, and very likely, begins to believe it may be within his reach to become one of the great men who have left their mark upon the world's history. The years go by, he competes in the examinations of school and college with his fellows, and soon finds his place among them. He knows that he can *beat* such and such of his companions, that there are others with whom he runs on *equal* terms, and others whose intellectual feats he cannot begin to approach, and it is an interesting fact in tracing back the pedigree of most of our great men, that those of high literary attainments seldom descend from parents very much their inferior, any more than great athletes descend from puny parents, or a swift race horse from a sluggish sire.

An interesting point, connected partly with physiological and partly with pathological transmission, is the fact that that peculiar, intangible something, which we call *longevity*, is a matter of inheritance. This fact is of importance to the individual in calculating his chances of life. It is often of great importance to the physician, in making up his estimate as to the chances of recovery in a patient, and it should be considered of much more importance than it is in the calculation of life insurance. One who can show that he comes from a long-lived stock ought to reap some benefit (other things being equal) over him who comes of a short-lived race. But it is correct to say that longevity does not depend upon that correct adjustment of the bodily functions which we call health, for it is a truth that many long lived people come of a positively invalid ancestry, whose weaknesses, no less than their longevity, they inherit. It is common to see those who have been sickly from their youth up and yet who have taken a long time to "burn out." They belong to that class of "chronic cases" on whom the old physician falls back at ease in those unfortunate periods of dullness that some times affect the sick market of his vicinity, and serve to afford him some solace in the absence of the more active maladies that afflict us.

On the other hand, it may be predicated of certain families that not many of them will live beyond a certain defined period. They are blown out easily as by a puff of air. The gusts of disease, that serve only to flicker the flame of life in others, extinguishes them utterly. No man engaged in the practice of medicine can have failed to notice how much easier the members of one family die from the same causes than those of an other.

Turgot said, as he neared his fiftieth year, that "he must put his house in order, as none of his family lived much beyond that period." Cognizant of this fact, he wrapped his cloak about him, and died in his fifty-second year.

While of any individual life we can predicate only the general uncertainty, yet of a family we may be assured (barring accidents) that a very definite portion of them will live to old age. They may possess what we call "tenacity of life." They withstand the shocks and buffets to which (averaging things) they *ought to* succumb, and continue on through the ills of existence, till we, who are younger and anxious to assume their duties, ask if they will not live always. But the happy old octogenarians continue on and live, many of them to stand by the open graves of many of us younger ones, who have not that tenacity with which they are endowed.

Thus far we have considered those physical transmissions which belong to the individual in a condition of health ; we pass from this to the pathological side of the subject, viz.: the tendency *to* and transmission *of* disease, and in doing this it is necessary to distinguish between "a tendency to disease," and *disease itself.* Generally speaking, *disease itself is not transmitted*, that is to say, a child is not actually *born* with consumption or scrofula, there is no tuberculous deposit in the lung, or scrofulous swelling *at birth*, nor perhaps for a very long period afterwards, but there is *the predisposition to it.* In the vast majority of cases we have the "*tendency to the disease*," rather than the disease itself, and in apoplexy, epilepsy, gout, rheumatism, insanity, etc., etc., we never have the disease itself at first, but we have the inherited constitution, the hereditary predisposition, out of which, sooner or later (if death does not come in at some other door), the diseases themselves will be developed. The principle is there, often remaining for a long time dormant, sometimes even passing over one generation to appear in the next; but there is a force, although it may be a latent force, that needs but the provocation of some untoward circumstance to fan it into activity, as the smouldering embers are kindled into a flame by a breath of air. *This* is what we *transmit* to those who come after us, an inheritance not to be desired.

The relation of the condition of the parent as *cause* and result in the child as *effect* is so apparent and so frequent that we are often enabled to predict of the members of a certain family, now in the bloom of health and exuberance of youth, that they will wither and die of the same diseases as those of their ancestors who preceded them.

Another point, worthy of our consideration in transmissions is the possibility of *acquired conditions* becoming hereditary. If we could trace back to the starting point, we should find that every hereditary taint had its origin in a vicious habit or excess of some of our ancestors, more or less remote, and that there *was* a time when those baneful influences did not exist, that the causes which tend to the production of these hereditary maladies are constantly at work, undermining the foundation of our mental and moral structure, that all excesses,both of mind and body, operated not only to impair the constitution of the individual who indulges in them, but are carried forward as baneful legacies to those who come after us. To live thus is to sin, not only against oneself, but against generations yet unborn. It is not only to perpetuate an evil that exists in us, but it is *originate* an evil, to cultivate it, and then to entail it upon those who follow after us. Notably under this head comes the matter of hereditary inebriety.

Time and space prevent me from opening to any extent into this vast field, but from personal observation I am lead to believe that not only is heredity *some times* a cause of drunkeness, but that it is the most potent cause we have. As the inherited predisposition to any other disease lurks hidden in the system for years, until at last by some untoward circumstance it suddenly and alarmingly makes its presence known, so the inherited appetite, of whose presence the victim may be entirely unaware, suddenly seizes him in an iron grasp, from which only the most resolute and determined efforts can free him.

In a recent lecture touching upon this subject, Dr. Joseph Cook of Boston says : "The *theory* that drunkenness is a disease is rapidly going out of fashion among experts." In this statement Dr. Cook plainly indicates his ignorance of the subject upon which he is speaking. The best authorities accept it, not as a *theory*, but as a *fact*. In the report of The Walnut Hill Asylum for the year 1878, we find the following statement : "Of the forty-two cases admitted during the year eight were cases where the disease was inherited from one of the parents, thirteen where it was inherited from both parents and grandparents," making just fifty per cent. where the appetite was clearly hereditary.

At the Ward's Island Asylum I gathered the following facts : "Of 100 patients made insane through drink, the fathers of thirty-two were drunkards, the mothers of twelve were addicted to drink, and in fourteen cases both parents and grandparents used liquor." Dr. T. D. Crothers, Superintendent of the Walnut Hill Asylum, says: "Inebriety is a physical, and usually an inherited disease, not a moral one, with cause, beginning, progress and decline,as well marked as intermittent fever." Dr. Robert Bird, late Surgeon-General in the British India Army, says : "Men get drunk from various reasons. One gets drunk to drown his sorrow, another because of his convivial qualities, another because he is weak minded, and is shamed into it by his stronger minded companions, a fourth because the traditions of his trade demand it, and a fifth *because an inherent quality of his nature demands it*," in other words, he gets drunk *because he cannot help it.* If we can remove the cause of the first one's sorrow, counteract the convivial qualities of the second, strengthen the weak mindedness of the third, and give the fourth different trade surroundings, we shall do much to counteract the evil, but what shall we do with the fifth, who has in him that *inherited appetite* for drink?" Dr. Cooke with many others, evidently misinterprets the true meaning of the word "drunkard." By "drunkard" we do not mean those persons who sometimes get drunk, being led into into it by some external conditions, or some peculiar surroundings. A *true drunkard* is he who cannot, or can only partialtially, control his appetite for strong drink. At the one extreme stands the chronic drunkard, who is continually drunk when he has the means of making himself so, and at the other that man, who, although he heretofore has, and is still, passing through life a sober man, is nevertheless a drunkard, *a latent one*, a man of strong mind and iron will, who knows that a little drink will let loose the caged devil within him, and is therefore, a total abstainer.

[TO BE CONTINUED.]

